# Fear of Vaginal Penetration in the Absence of Pain as a Separate Category of Female Sexual Dysfunction: A Conceptual Overview

**DOI:** 10.5041/RMMJ.10293

**Published:** 2017-04-28

**Authors:** David Rabinowitz, Lior Lowenstein, Ilan Gruenwald

**Affiliations:** Department of Obstetrics and Gynecology, Rambam Health Care Campus, Haifa, Israel; and The Bruce Rappaport Faculty of Medicine, Technion–Israel Institute of Technology, Haifa, Israel

**Keywords:** Avoidance, fear, female sexual dysfunction, pain, penetration phobia, sexual pain, vaginismus

## Abstract

Functional sexual pain disorders in women are a particular challenge to the gynecologist, inasmuch as phobic avoidance and guarding on the part of the patient lead to difficulties in the gynecological examination and diagnosis. In some such cases examination may even be impossible. Vaginismus is the commonly diagnosed etiology of such cases. This article offers an overview of vaginismus and approaches to its treatment but also examines a subset of penetration-avoidant patients who do not appear to have a pain component. We have reviewed this separate category conceptually and clinically, and propose that this case subset be separated from the diagnosis of vaginismus and designated as vaginal penetration phobia (VPP).

We further propose that this category be diagnosed as one of several possible presentations of phobic disorder, under the rubric of mental health disorder, and thus be separated from gynecology. The nosological implications are raised.

## INTRODUCTION

Female sexual dysfunction (FSD) may present as disorders of sexual desire, arousal, or orgasm, alone or in combination, and separately as sexual pain disorders, a category which is independent of the triphasic sexual response cycle. A significant modification of the former category in the *Diagnostic and Statistical Manual of Mental Disorders, 5th ed.* (DSM-5), is the consolidation of the first two elements into a composite “female sexual interest/ arousal disorder,”^1^ while sexual pain disorders remained a distinct category.

The sexual pain disorders are subdivided into two distinct groupings following the DSM-5: dyspa-reunia and vaginismus. Estimates of the overall prevalence of dyspareunia are in the range of 6%–45% depending on age, and for vaginismus the range is 1%–6%.^2^ Dyspareunia refers to pain syndromes resulting from gynecological pathology and will not be discussed here. Vaginismus is a function-al pain disorder related to painful hypertonic levator muscles as a response to pending vaginal penetration in any form, and associated with extreme anxiety and avoidance of vaginal penetration. There is some debate over the role of spasm or hypertonia in the vaginal musculature in vaginismus.^3^

In recent years, we have identified a relatively under-reported problem in certain patients visiting our sexual dysfunction clinic (SDC): the fear of vaginal penetration in the absence of any clear-cut pain component. In this paper we aim to identify the main distinct features of this clinical phenomenon, and thereby to separate it from other sexual pain disorders, especially vaginismus. We furthermore attempt to identify its conceptual underpinnings, and the implications thereof. In order to elucidate the features of this separate syndrome, the concept of vaginismus will be briefly reviewed, to which the separate syndrome can be compared.

## VAGINISMUS

Women who present with a complaint of a lifelong inability to tolerate vaginal penetration, most commonly in the younger age group, are a diagnostic and therapeutic challenge for the physician. Diagnostic considerations commonly assume vaginismus, as defined above, by default. A secondary fulminating panic-like phobic avoidance completes the picture. Vaginismus is primarily a genital pain syndrome with secondary behavioral features.

The latest *Diagnostic and Statistical Manual* of the American Psychiatric Association (DSM-5), replaced the term “vaginismus” with the diagnostic category “genito-pelvic pain/penetration disorder,” thus introducing some degree of flexibility in approaching this disorder. Two of four conditions need to be present to make this diagnosis: difficulty in having intercourse, genito-pelvic pain, fear of pain or penetration, and tension of the pelvic floor muscles. While it is formally possible to make this diagnosis according to these criteria even without the pain factor, most of the discussion in the relevant DSM section refers to the pain factor as a central concept.^1^,^4^ A useful discussion on the role of pain in this condition is found in the paper by Reissing et al.^5^

It is the secondary phobic anxiety which defines the primary clinical presentation. This is the element that prevents the internal gynecological examination. The woman will experience instant fulminating anxiety, often terror, with any approach to penetration, resulting in vigorous *avoidance* behaviors such as pulling away or legs tightly pressed together, intense rapid fear arousal, and vocal reactions with refusal to permit penetration. Interestingly, relatively few report any past history of sexual or other trauma. Conversely, pre-existing sexual trauma does not invariably predict this condition. Vaginismus generally appears to develop *de novo* and without clear etiological antecedents. It is usually a primary condition. Secondary vaginismus developing after a pre-existing normal penetrative capacity is best seen as a result of physical pathology until proven otherwise.

Avoidance behavior is primarily a defensive mechanism aimed at preventing and avoiding intolerable expected pain, but avoidance should not be underestimated as it also plays a central role in the continuation of the condition. As in all phobic disorders, the woman is constantly guarding against any penetration, and will not dare to insert tampons, or permit inadvertent penetration, even self-digital, during erotic activity, thus preventing any form of naturalistic behavioral desensitization. This ensures the full expression of the condition with each threat of perceived exposure—in this case, vaginal penetration.

Interestingly, clinical experience shows that some sexual partners may be able to accept the partner’s avoidance behavior, and co-operate by developing a non-penetrative sexual life. They are often noted to be very concerned about causing pain to their partners, and in that sense they become participants of the avoidance behavior themselves. Their admirable “gentlemanly” understanding and behavior paradoxically contributes to the long-term stability of the condition, inasmuch as there is no demand for change. It is not uncommon for such couples to present for help only when they feel ready to have a child and are now faced with a major hurdle. While some women may overcome this barrier and conceive spontaneously, others may choose to have intrauterine insemination. The mode of delivery is another dilemma; often women with vaginismus may choose to have cesarean section in order to avoid vaginal procedures during the delivery process.

Women suffering from vaginismus are generally noted to have an otherwise normal sexual response. They may surprisingly have satisfying intimate relationships, and may report normative attraction to their partners. Certainly the issue here is not sexual phobia, defined as a phobic reaction to and avoidance of arousal or orgasm, or to the natural secretions of sex (semen or vaginal lubrication).^6^ Of further interest is that vaginismus is rarely encountered in lesbian patients, or its functional equivalent in homosexual males whose partners want anal sex. Implicit in the vaginismus discourse is that it is a phenomenon found essentially in heterosexual women.

Diagnosis is made optimally by a gynecological examination, but this is riddled with contradiction. Only by gynecological examination can painful peri-vaginal hypertonus be definitively diagnosed and differentiated from other types of gynecological pain, e.g. vulvar vestibulitis, or other covert gynecological pathology, hymenal or otherwise.^7^ However, it is quite usual for the gynecologist to defer with a full gynecological examination given the realities of the condition, relying on the history given by the patient^8^ and the observed behavior in the examination attempt. Ultrasound and other non-invasive gynecological procedures are relied upon to exclude anatomical pathology. Unfortunately, often transvaginal ultrasound cannot be done, and abdominal ultrasound has a limited diagnostic value. Not uncommonly, women with vaginismus try to evade a visit to a gynecologist, as part of the overall avoidance pattern. However, a successful gynecological examination may in fact reveal that the area is not painful, yet the phobic reaction to intimate penetration results in muscle contraction producing dyspareunia and avoidance behavior.

## FEAR OF VAGINAL PENETRATION: VAGINAL PENETRATION PHOBIA

This brief review of vaginismus prepares the ground for a broader understanding of the general problem of women who fear and avoid vaginal penetration. The point that we wish to make is that phobic avoidance of vaginal penetration is not always vaginismus, or pain-based. There appears to be a subset of women presenting with penetration phobia who actually report a history of at least one gynecological examination, perhaps partial at best, involving some degree of digital penetration, or possibly other forms of vaginal penetration, *in which no pain was experienced*. This fact must raise the question as to whether the automatic assumption that vulvo-vaginal pain of any kind lies at the core of every case of penetration avoidance.

These women otherwise show a full-blown phobic avoidance picture, to all extents and purposes indistinguishable from the phobic pattern as described above in vaginismus. In the clinical setting they tend to be younger women, single or living in a couple relationship as described above. Presentation to the clinical service for help may be, as for vaginismus, related to the desire for full sexual intercourse, or the desire for pregnancy in a couple with a formally unconsummated relationship. A less obvious component of the motivation for help lies in the experience of what may be termed “anticipatory anxiety,” inasmuch as these patients live with a subliminal background fear of the next intimate encounter and are unable fully to relax in approaching intimacy. Anticipatory anxiety (the fear of another panic attack) is in fact a well described feature of panic disorder and plays an important role in the disruption of the quality of life.^1^

The full implications of this distinction have not received much attention in the literature.^2^,^9^,^10^ In one study,^10^ vaginal penetration phobia (VPP) is separately identified, but remains conceptually tied to vaginismus. Here we imply that penetration phobia without pain constitutes a subtype of pure phobic disorder and should not fall under the diagnostic category of vaginismus. This view is similar to that expressed by Vonk et al. in a single case study.^9^ It is a condition most correctly treated primarily by mental health professionals specializing in cognitive and behavioral techniques, and may require the additional component of antipanic psychopharmacology.^6^ In practice the mental health professional may recruit gynecological input to the treatment regimen as the patient may benefit from graded approaches to a gynecological examination or dilators under gynecological supervision; however, this may be offered only after a full program of cognitive behavioral therapy has prepared the patient for the “gynecological” stage of treatment.

At this point the core differences between vaginismus and what may be termed “penetration phobia” can be summed up: in vaginismus the phobic avoidance behavior protects against expected intolerable pain, while in penetration phobia the phobic avoidance behavior protects against fulminating panic anxiety. This form of anxiety, bordering on terror, is no less intolerable than pain. It only requires imagining a patient with severe claustrophobia who is trapped in an elevator; such an individual will rather climb 20 stories all his life only to avoid any such event, ever. This is an example of avoidance behavior, which affects quality of life. To complete the picture, at the core of the under-standing of the phobic disorders in general is that the fulminating panic anxiety occurring on exposure to the phobic stimulus is essentially irrational;^1^ it is the experiencing of severe anxiety when exposed to a stimulus that is not inherently and invariably anxiety-provoking. This may include butterflies, elevators, open spaces, dolls—and pending vaginal penetration in the absence of pain.

The importance of this distinction, between vaginismus and penetration phobia, is that the former is located in the domain of clinical gynecology, albeit “psychosomatic” gynecology, while the latter is located in the domain of clinical psychiatry, appearing to be a *bone fide* variant of phobic disorder. Ensuring that the patient gets to the right specialist quickly promises a more effective and rapid treatment environment, depending on effective triage by the gynecologist at first examination.

In patients who are unable to co-operate with the gynecologist, the central problem is to make the diagnosis. Where internal gynecological examination is possible, even in a limited way, a pain factor may be detected which essentially clinches the diagnosis of vaginismus. However, in some cases the clinician may base his diagnosis on a history of past gynecological examination in which pain was or was not excluded, depending on the ability of the patient to co-operate in the past. Diagnostic uncertainty may well be the starting-point if an attempt at gynecological examination fails or is refused, and there is an absence of any history of past gynecological examination.

## TREATMENT

Patients may enter treatment in one of two groups: those with a definitive diagnosis and those without. A clear diagnosis is important in order to decide on the therapeutic path. If vaginismus is the primary diagnosis, then treatment in principle begins with the reduction of avoidance behavior, in various settings. This may be achieved by gentle desensitization in the gynecological setting by a doctor or physical therapist who gradually exposes the patient to approaching digital penetration using support, relaxation exercises, and often a mirror to allow the patient a visual tracking of the treatment in the lithotomy position. This approach is probably uncommon as it is time-consuming. Alternatively the patient may be offered detailed explanation and support and be given dilators and instruction to use at home (often starting the therapy with the physical therapist in the clinic followed by home exercise with dilators). Mental health therapists specializing in behavioral and cognitive therapies may be recruited by the gynecologist to set up a systematic desensitization schedule designed ultimately to facilitate digital or dilator penetration by the gynecologist. While such cases present less commonly to mental health professionals, mostly they are managed in gynecology clinics, and the responsibility for care thus remains in this setting.

Sexologists who used to manage vaginismus more in the past have noted that the prognosis of treatment is substantially improved if it is carried out in the presence of the patient’s sexual partner. Given full patient consent and co-operation, he may be recruited by the treating physician-sexologist or physical therapist to perform digital penetration in the office as part of the treatment program, with prior negotiation and the correct timing. The partner thus undergoes a learning experience that addresses his own hesitancy, lack of confidence, and fear of hurting his female partner. In a sense this places him in the position of “co-therapist,” thus enhancing the transfer of treatment gains from the consulting room to the bedroom. This approach will not be suitable for all couples and not for all physicians, and the partner may then be recruited by the patient in the privacy of the home setting, one way or another, but forfeiting the benefits of immediacy and real-time learning in the clinical setting.

If the diagnosis is not definitive, it may be treatment-emergent. Whether some have pain, or not, will declare itself in the course of treatment, requiring re-diagnosis and “course corrections” by the treating professional. Thus if the final diagnosis is VPP, cognitive behavioral therapy (CBT), with or without anti-panic medication, may be sufficient.^6^ The patient may well be able to achieve and tolerate vaginal penetration without gynecological intervention. However, should the diagnosis emerge as vaginismus, gynecological intervention is essential, although prior CBT may be helpful as facilitation.

Thus a multidisciplinary approach in a polyclinic may sometimes offer a better solution for those women, in order to reach a more accurate diagnosis and to set a better treatment plan.

An intriguing theoretical consideration can be taken into account at this point: that perhaps all forms of penetration phobia are primarily phobic disorders, of which only some have the psycho-physiological factor of increased perivaginal tone and pain on penetration attempts. This fits with the pragmatics of the treatment approach, inasmuch as all such cases are treated from the outset with the focus on avoidance behaviors. What this requires is further clinical research on patient populations. The objective will be to determine to what extent the distinction between two groups as described above, as an emergent hypothesis derived from the clinic, is supported by further studies.

## SUMMARY

This unique complaint stands apart from commonly known categories of sexual dysfunction. Due to its seemingly relatively low prevalence, fear of vaginal penetration in the absence of pain needs to be more recognized. Once detected and diagnosed, cognitive and behavioral treatment is very effective and could prevent years of abstinence from wanted sexual enjoyment and satisfaction. Multimodal treatment regimens need to be deployed, and a multidisci-plinary approach is usually necessary.

## Abbreviations

CBT: cognitive behavioral therapy
FSD: female sexual dysfunction
SDC: sexual dysfunction clinic
VPP: vaginal penetration phobia

## References

[1] American Psychiatric Association (2013). Diagnostic and Statistical Manual of Mental Disorders.

[2] van Lankveld JJ, Granot M, Weijmar Schultz WC (2010). Women’s sexual pain disorders. J Sex Med.

[3] Reissing ED, Binik YM, Khalifé S, Cohen D, Amsel R (2004). Vaginal spasm, pain, and behavior: an empirical investigation of the diagnosis of vaginismus. Arch Sex Behav.

[4] Sadock BJ, Sadock VA, Ruiz P (2015). Kaplan and Sadock’s Synopsis of Psychiatry.

[5] Reissing ED, Binik YM, Khalife S (1999). Does vaginismus exist? A critical review of the literature. J Nerv Ment Dis.

[6] Kaplan HS, Fyer AJ, Novick A (1982). The treatment of sexual phobias: the combined use of antipanic medication and sex therapy. J Sex Marital Ther.

[7] Huber JD, Pukall CF, Boyer SC, Reissing ED, Chamberlain SM (2009). “Just relax”: physicians’ experiences with women who are difficult or impossible to examine gynecologically. J Sex Med.

[8] Reissing ED (2012). Consultation and treatment history and causal attributions in an online sample of women with lifelong and acquired vaginismus. J Sex Med.

[9] Vonk ME, Thyer BA (1995). Exposure therapy in the treatment of vaginal penetration phobia: a single-case evaluation. J Behav Ther Exp Psychiatry.

[10] Muammar T, McWalter P, Alkhenizan A, Shoukri M, Gabr A, Bin AA (2015). Management of vaginal penetration phobia in Arab women: a retrospective study. Ann Saudi Med.

